# Exploratory Study of Tegaserod for Dyspepsia in Women Receiving PPIs for Heartburn

**DOI:** 10.1111/j.1753-5174.2008.00012.x

**Published:** 2008-12

**Authors:** Nimish Vakil, Farid Kianifard, Ivan Bottoli

**Affiliations:** * University of Wisconsin School of Medicine and Public Health, Madison, Wisconsin and Marquette University College of Health SciencesMilwaukee, WI, USA; † Novartis Pharmaceuticals CorporationEast Hanover, NJ, USA

**Keywords:** Clinical Trial, Dyspepsia, Gastrointestinal Transit, Heartburn, Tegaserod

## Abstract

**Background and Aims:**

Tegaserod is a selective serotonin receptor (5-HT_4_) agonist that relieves dysmotility symptoms associated with constipation. Here we explore its effects on functional dyspepsia symptoms and heartburn during continued proton pump inhibitor (PPI) treatment.

**Methods:**

In this multicenter pilot study, following a 2-week screening/baseline period, women with functional dyspepsia and persisting heartburn treated with PPIs received add-on open-label tegaserod 6 mg twice daily (bid) for 4 weeks. Treatment responders were then randomized 1:1 to continue double-blind tegaserod or placebo therapy for 6 weeks. Efficacy variables included the proportion of days with satisfactory relief of dyspepsia symptoms (early satiety, postprandial fullness and bloating) as well as the change in individual symptom severity scores for these three cardinal dyspepsia symptoms. Health-related quality of life was evaluated using a validated questionnaire, the Nepean Dyspepsia Index. Adverse events (AEs) were monitored.

**Results:**

Of 101 women enrolled, 71 completed open-label treatment, and 70 responders were randomized to double-blind treatment. The proportion of days with satisfactory relief of dyspepsia symptoms (least squares mean, LSM) increased with tegaserod and placebo, to 0.69 and 0.62, respectively at study end (*P* = 0.366). Similarly, both groups showed improvements in the composite daily symptom severity score (overall LSM change from baseline of 1.55 and 1.57, *P* = 0.934), and the Nepean Dyspepsia Index (overall LSM change of −39.0 and −37.8, *P* = 0.537). Tegaserod was well tolerated. Diarrhea was the most common AE (8.1% tegaserod, 0% placebo). There were no serious AEs or deaths.

**Conclusions:**

A significant treatment effect was not demonstrated in this study using a treatment-withdrawal methodology. In future studies of functional dyspepsia patients with heartburn, a more rigorous parallel-group study design should be considered.

## Introduction

Symptoms of heartburn and/or dyspepsia are reported by an estimated 10–40% of the population in Western countries [1–3]. Dyspepsia refers to a group of symptoms (postprandial fullness, early satiety, epigastric pain, epigastric burning, bloating) that are considered by most physicians to originate from the gastroduodenal region of the stomach and is further defined as “functional dyspepsia” if standard investigations do not provide an explanation for symptoms. Heartburn is defined by the presence of a retrosternal burning sensation, which tends to move cephalad into the neck. Functional heartburn is diagnosed when the heartburn is not accompanied by evidence of gastroesophageal reflux disease (GERD) as evaluated by endoscopy or 24-hour esophageal pH measurement. Functional heartburn may occur concomitantly with dyspepsia symptoms and such overlap has been illustrated in a study of gastrointestinal (GI) symptoms in 67 patients with functional heartburn. Sixty-four percent of functional heartburn patients in this study had upper abdominal pain, 80% had upper abdominal discomfort, and 60% had early satiety [4].

Little is known about the pathophysiology of the overlapping symptoms of functional dyspepsia and functional heartburn. Consequently, the most appropriate therapeutic targets have yet to be defined, and current treatment options are limited. Proton pump inhibitors (PPIs) are often prescribed specifically for the relief of heartburn and upper abdominal pain. Although predictable improvements in these two symptoms have been demonstrated in acid-related conditions, PPI therapy appears to offer little benefit for patients with functional dyspepsia and/or functional heartburn [5,6].

Tegaserod is a selective serotonin type 4 receptor (5-HT_4_) agonist that has been shown to relieve dysmotility symptoms associated with irritable bowel syndrome with constipation (IBS-C) and chronic idiopathic constipation [7,7–11]. Furthermore, clinical trials have shown that tegaserod enhances gastric emptying, accelerates GI transit in healthy subjects [12], normalizes gastric emptying in functional dyspepsia patients with delayed gastric emptying [13], and increases gastric accommodation and compliance [14,15]. As most functional dyspepsia studies to date have focused on measuring the pharmacodynamic effects of tegaserod alone, there are very limited data available regarding the effects of this agent on symptom improvement and other patient-assessed outcomes. This is particularly true for functional dyspepsia patients with overlapping functional heartburn who are already receiving PPI treatment.

Therefore, a pilot study was designed to explore the possible effects of treatment with tegaserod 6 mg twice daily (bid) vs. placebo in women with overlapping symptoms of functional dyspepsia and functional heartburn who had received unsuccessful PPI treatment for their dyspeptic symptoms.

## Methods

### Study Design

**Figure 1:**
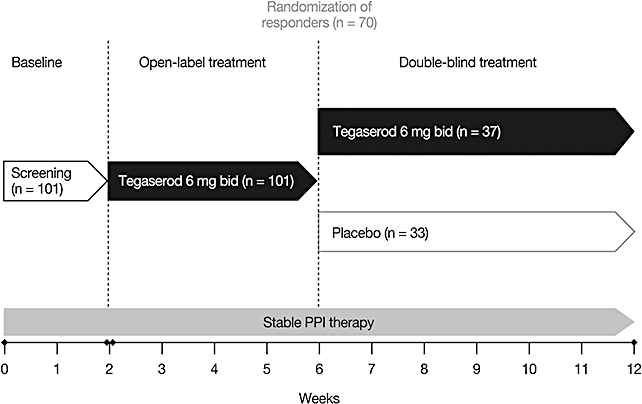
Study design.

This was a 12-week exploratory multicenter pilot study of women with functional dyspepsia and functional heartburn receiving PPIs whose functional dyspepsia symptoms responded to open-label tegaserod treatment. Responders to open-label tegaserod treatment were randomized to double-blind, placebo-controlled withdrawal of tegaserod while PPI treatment was continued (Figure 1).

All subjects were required to have had a negative upper GI endoscopy performed within the preceding 24 months at a time when their functional dyspepsia and functional heartburn symptoms were active. Following a 2-week screening/baseline assessment phase, all eligible patients received open-label treatment with tegaserod 6 mg bid for 4 weeks, which was added to their current PPI regimen. Treatment responders were identified based on their response during the last week of open-label treatment to a weekly Global Symptom Assessment question. Tegaserod responders were then randomized 1:1 to receive tegaserod 6 mg bid or placebo bid for 6 weeks, i.e., the patients either continued to receive tegaserod/PPI therapy or were switched to placebo/PPI therapy. Documentation confirming the continuation of a stable PPI therapy regimen was required throughout the study in both groups.

Randomization assignment was performed by the study sponsor using a validated system that automates the random assignment of the subjects to the two treatment groups. The randomization scheme and the randomization number code for individual patients was locked after approval by a Biostatistics Quality Assurance Group and remained confidential until required for data analysis following study completion. Patients and study investigators remained blinded to treatment throughout the study. Blinding was maintained using placebo and tegaserod tablets of identical appearance and packaging the tablets in containers that were also identical in appearance.

The study protocol was approved by an Institutional Review Board/Independent Ethics Committee/Research Ethics Board at each participating center and performed according to the Declaration of Helsinki and the principles of Good Clinical Practice. All patients gave written informed consent before participating in the study. The study is registered under the ClinicalTrials. gov number: NCT00171470.

### Patient Population

Women (≥18 years) receiving PPI therapy at stable doses for at least 4 weeks for heartburn were enrolled in this study if they had self-reported symptoms of functional dyspepsia (mid–upper abdominal discomfort associated with post-meal fullness, early satiety while eating and bloating, as well as abdominal pain, nausea, vomiting, regurgitation or constipation) for at least 12 weeks (not necessarily consecutive) during the previous 12 months (Rome II criteria for functional dyspepsia) [16]. Starting at screening/baseline and continuing throughout the study, patients rated their discomfort caused by their functional dyspepsia symptoms on a daily basis using a 7-point scale (0 = no discomfort at all, 1 = minimal discomfort, 2 = mild discomfort, 3 = moderate discomfort, 4 = moderately severe discomfort, 5 = severe discomfort, 6 = very severe discomfort). In order to qualify for inclusion, patients’ average symptom severity score was required to be at least “mild” for two or more of the following functional dyspepsia symptoms: post-meal fullness, early satiety while eating, and bloating. To enter the study, patients were also required to report a lack of satisfactory relief of dyspeptic symptoms during both of the 2 weeks of screening/baseline. This was based on a response of “No” to the weekly Global Symptom Assessment question:

Over the past week, did you have satisfactory relief of your mid–upper abdominal discomfort which may include early fullness (early satiety) while eating, post-meal fullness, or bloating? (Yes or No)

Key exclusion criteria were a history of erosive esophagitis or peptic ulcer disease, intestinal obstruction, symptomatic gallbladder disease, sphincter of Oddi dysfunction, or abdominal adhesions; a history of major abdominal surgery affecting GI anatomy; evidence of acute or serious medical conditions other than dyspepsia or evidence that dyspeptic symptoms are relieved by defecation and/or associated with a change in stool frequency or stool form. Heartburn as the current most bothersome symptom also resulted in exclusion from the study, as did receiving ulcer prevention treatment with acid suppressive therapy. A history of frequent or chronic diarrhea, or treatment for *Helicobacter Pylori* within the previous 6 months, were also exclusions. In addition, pregnant or breastfeeding women, and those of childbearing age who were not using an approved method of contraception, were excluded. Prior treatment with medications that could mask the effect of the trial medication was disallowed. These medications included systemic cholinergics and anticholinergics (e.g., l-hyoscyamine, clidinium, dicyclomine), calcium channel blockers (e.g., verapamil, amlodipine), narcotic analgesics, nitroglycerin derivatives, prokinetics (e.g., metoclopramide), macrolide antibiotics, and histamine H_2_ receptor antagonists. Patients were permitted rescue medication with sodium bicarbonate, sodium alginate, and/or calcium carbonate.

### Efficacy Assessments

Patients completed diaries throughout the three study phases (screening/baseline, open-label and double-blind). During the screening/baseline phase they recorded their responses to the following Global Symptom Assessment question on a weekly basis:

Over the past week, did you have satisfactory relief of your mid–upper abdominal discomfort which may include early fullness while eating, post-meal fullness, or bloating? (Yes or No)

During the screening/baseline and double-blind phases, a daily diary entry was required for a similar Global Symptom Assessment question:

Today, did you have satisfactory relief of your mid–upper abdominal discomfort which may include early fullness while eating, postprandial fullness, or bloating? (Yes or No)

In their daily diary, patients also recorded the severity of their individual symptoms. This was a daily occurrence throughout all three study phases to quantitate each patient's assessment of the discomfort caused by the specific functional dyspepsia and functional heartburn symptoms (early satiety while eating, post-meal fullness, bloating, abdominal pain, nausea, heartburn, regurgitation, and constipation). The symptom assessment was captured using a 7-point scale (Rome II criteria) [16] which ranged from a score of 0 = no discomfort at all to a score of 6 = very severe discomfort.

To be entered into the study, patients had to answer “No” to the weekly Global Symptom Assessment question for both weeks of the 2-week screening/baseline phase. After study entry, the primary efficacy variable was the proportion of days with satisfactory relief of dyspepsia during the double-blind phase based on the daily Global Symptom Assessment question. This was defined as the number of days in which the patient responded “Yes” to the Global Symptom Assessment question divided by the number of days the question was answered. Secondary variables included the average daily severity score of three key symptoms during the double-blind phase (early satiety while eating, post-meal fullness and bloating). Symptoms were assessed both as a daily composite score for the three key functional dyspepsia symptoms (mean severity value across all three symptoms), and as average daily scores for the individual symptoms (only daily composite functional dyspepsia symptom score data are reported here).

Patients were also asked to complete a validated, disease-specific quality of life instrument, the Short Form-Nepean Dyspepsia Index questionnaire (SF-NDI) [17,18]. This was done at each study visit (Day 0, Day 29, Day 50, and Day 71 or early discontinuation).

### Safety Assessments

All adverse events (AEs) were recorded and monitored. An AE was defined as any undesirable sign, symptom, or medical condition occurring after starting study drug even if the event was not considered to be related to study drug. In addition, patients’ vital signs, blood chemistry and hematology were measured and electrocardiogram (ECG) evaluations were performed at screening/baseline and end of study.

### Statistical Analyses

All statistical tests were conducted against a two-sided alternative hypothesis, employing a significance level of 0.05.

The intent-to-treat (ITT) population comprised all randomized patients who received at least one dose of study drug post-randomization and had at least one post-baseline assessment of the primary efficacy variable. The safety population, comprising all patients who received at least one dose of study drug, was used for all safety analyses.

The primary efficacy variable was analyzed for the ITT population using an analysis of covariance (ANCOVA) model that included treatment, center, and proportion of days with satisfactory relief during screening/baseline as explanatory variables. Least squares mean (LSM) values, LSM treatment differences and 95% confidence intervals (CI) for the difference in the two treatments were reported, based on the fitted linear model. To assess the impact of missing data on the proportion of days with satisfactory relief, a sensitivity analysis was performed which imputed “non-response” for patients who did not complete at least half (21 days) of the assessments. In addition to evaluating the effect over the entire double-blind treatment phase, the primary efficacy variable was analyzed similarly for each study week.

The analysis of average daily severity scores for early satiety while eating, post-meal fullness, and bloating was similar to that for the primary efficacy variable. The three symptom severity scores were averaged on a daily basis and then a grand mean value was calculated using all available data during the double-blind treatment phase.

Change from baseline in the total score and each domain score of the SF-NDI was analyzed using an ANCOVA model that included center and baseline as explanatory variables.

The sample size for this pilot study was not based on statistical power considerations. All statistical analyses were performed using SAS software, Version 8.2.

## Results

### Patients

**Figure 2:**
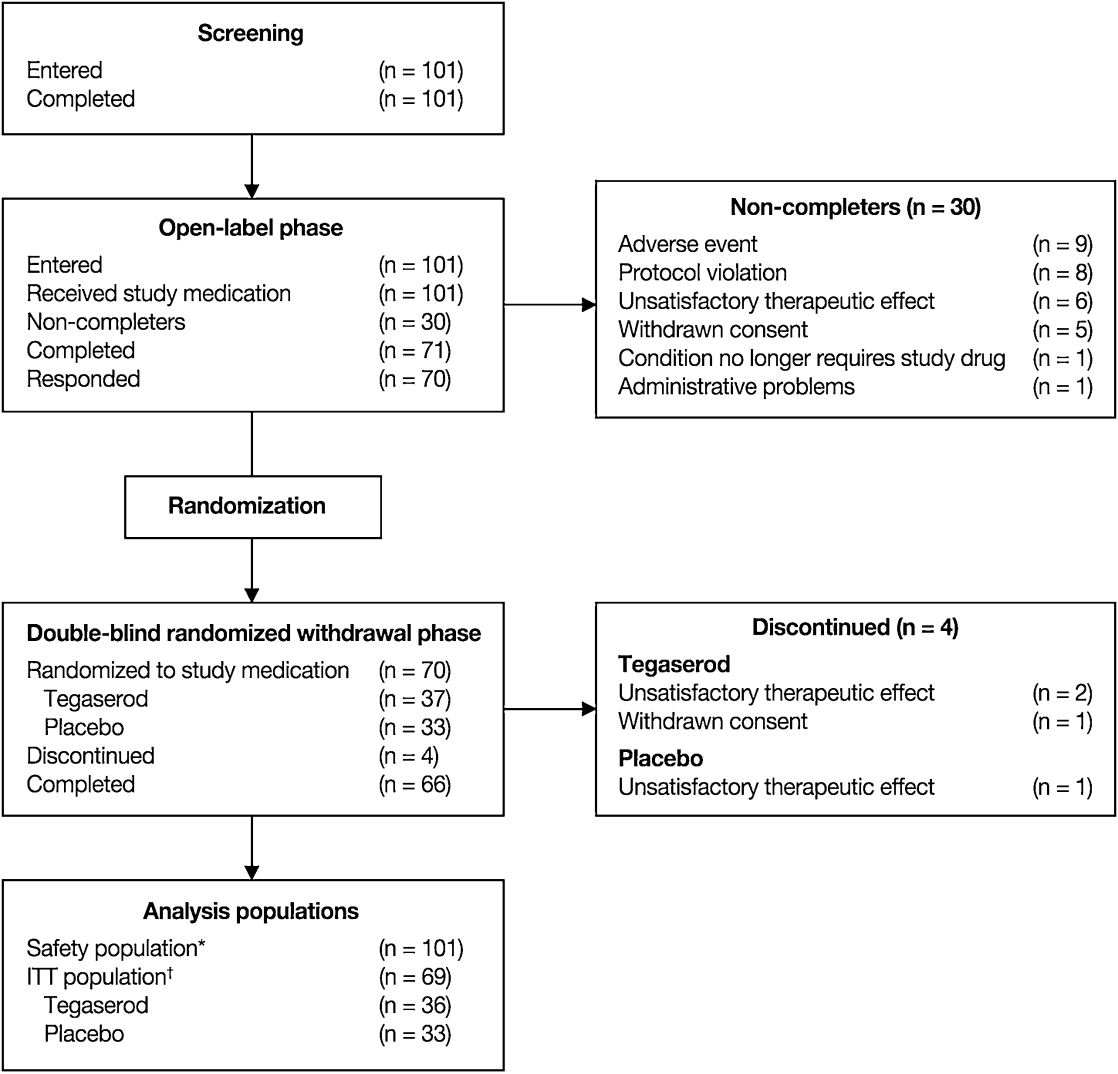
Summary of patient flow. *Received at least one dose of study medication. ^†^Received at least one dose of study medication post-randomization and had at least one post-baseline assessment of primary efficacy variable.

A total of 101 patients receiving PPI therapy qualified for and entered the study. Of these patients, 71 completed open-label treatment of whom 70 were judged to meet the criteria for study randomization as tegaserod responders and thus entered the double-blind treatment phase (Figure 2). The main reasons for discontinuation during the open-label treatment phase were AEs (n = 9), protocol violations (n = 8), and unsatisfactory therapeutic effect (n = 6).

Baseline demographics of the populations randomized to the two treatments were comparable and their characteristics were consistent with those of the overall study population (Table 1). As may be expected following selection of an enriched population of treatment responders, there were few discontinuations during the double-blind phase (n = 4). The discontinuations were due to unsatisfactory therapeutic effect (tegaserod n = 2 and placebo n = 1) and withdrawal of consent (tegaserod n = 1).

**Table 1:** Baseline patient characteristics

	All patients in open-label phase	Patients randomized to double-blind treatment	
		
Variable	Tegaserod	Tegaserod	Placebo
	(N = 101)	(N = 37)	(N = 33)
Age, years			
Mean ± SD	48.6 ± 14.8	47.9 ± 14.3	48.7 ± 16.1
Minimum, maximum	18, 80	19, 80	18, 73
Ethnic origin, n (%)			
Caucasian	71 (70.3)	27 (73.0)	23 (69.7)
African American	6 (5.9)	2 (5.4)	2 (6.1)
Asian	12 (11.9)	5 (13.5)	3 (9.1)
Other	12 (11.9)	3 (8.1)	5 (15.2)
Most common GI disorders,* n (%)			
GERD	52 (51.5)	23 (62.2)	15 (45.5)
Dyspepsia	49 (48.5)	16 (43.2)	17 (51.5)
Hiatus hernia	30 (29.7)	9 (24.3)	11 (33.3)
Constipation	22 (21.8)	9 (24.3)	9 (27.3)
Hemorrhoids	13 (12.9)	5 (13.5)	3 (9.1)
Other common conditions,† n (%)			
Hysterectomy	30 (29.7)	9 (24.3)	12 (36.4)
Asthma	22 (21.8)	4 (10.8)	10 (30.3)
Hypertension	19 (18.8)	7 (18.9)	7 (21.2)
Headache	18 (17.8)	7 (18.9)	7 (21.2)
Drug hypersensitivity	16 (15.8)	8 (21.6)	3 (9.1)
Cholecystectomy	14 (13.9)	3 (8.1)	7 (21.2)
Osteoarthritis	9 (8.9)	0	7 (21.2)

* Reported by ≥10% of patients in any group.

† Reported by ≥20% of patients in any group.

### Efficacy

#### Days with Satisfactory Relief of Functional Dyspepsia Symptoms

The LSM proportion of days with satisfactory relief of functional dyspepsia symptoms during the double-blind phase for the ITT population was 0.69 in the tegaserod group vs. 0.62 in the placebo group (*P* = 0.366; Table 2). This difference was not statistically significant. Evaluation of the proportion of days with satisfactory relief for the ITT population during each separate study week also showed no significant differences between groups, with LSM treatment differences at each week ranging from −0.028 to 0.127 (Table 2). Table 3 shows the effect of double-blind treatment on dyspepsia symptoms for the primary efficacy variable, the proportion of days with satisfactory relief of symptoms, indicating that there were negligible differences between the tegaserod and placebo groups.

**Table 2:** Effect of double-blind treatment on dyspepsia symptoms in women receiving PPI therapy: primary efficacy variable (ITT population*)

	Least squares mean				
			
	Tegaserod† (N = 36)	Placebo† (N = 33)	Treatment difference	*P* value	95% CI for treatment difference
Proportion of days with satisfactory relief‡ of symptoms (ALL WEEKS)	0.69	0.62	0.069	0.366	(−0.083, 0.222)
Start of double-blind phase					
Week 7	0.73	0.62	0.110	0.201	(−0.060, 0.279)
Week 8	0.73	0.61	0.127	0.181	(−0.061, 0.315)
Week 9	0.70	0.60	0.100	0.260	(−0.076, 0.275)
Week 10	0.68	0.60	0.087	0.341	(−0.095, 0.269)
Week 11	0.66	0.65	0.001	0.989	(−0.181, 0.184)
End of double-blind phase					
Week 12	0.69	0.72	−0.028	0.773	(−0.222, 0.166)

* Received at least one dose of study medication post-randomization and had at least one post-baseline assessment of primary efficacy variable.

† Estimated by analysis of covariance adjusted for center and baseline values.

‡ Defined as a response of “Yes” to the weekly Global Symptom Assessment question: “*Over the past week, did you have satisfactory relief of your mid–upper abdominal discomfort which may include early fullness (early satiety) while eating, post-meal fullness, or bloating?*”

**Table 3:** Effect of double-blind treatment on dyspepsia symptoms in women receiving PPI therapy: primary efficacy variable (mean ± SD; ITT population*)

	Proportion of days with satisfactory relief† of symptoms	Tegaserod (N = 36)	Placebo (N = 33)
Baseline	Week 1	0.07 ± 0.15	0.11 ± 0.19
	Week 2	0.07 ± 0.16	0.06 ± 0.16
Open-label phase	Week 3	0.44 ± 0.36	0.47 ± 0.38
	Week 6	0.71 ± 0.37	0.86 ± 0.26
Double-blind phase	Week 7	0.75 ± 0.32	0.63 ± 0.35
	Week 12	0.74 ± 0.37	0.77 ± 0.32

* Received at least one dose of study medication post-randomization and had at least one post-baseline assessment of primary efficacy variable.

† Defined as a response of “Yes” to the weekly Global Symptom Assessment question: “*Over the past week, did you have satisfactory relief of your mid–upper abdominal discomfort which may include early fullness (early satiety) while eating, post-meal fullness, or bloating?*”

#### Composite Daily Symptom Severity Score

In line with the findings observed for the primary efficacy variable, the combined average daily severity score of the three key symptoms (early satiety while eating, post-meal fullness, and bloating) for the ITT population during the double-blind phase did not differ significantly between treatment groups (Table 2). The LSM average daily severity score was 1.55 in the tegaserod group and 1.57 in the placebo group, representing a LSM treatment difference of −0.017 (*P* = 0.934). Similar results were observed for each of the three key symptoms when analyzed separately.

#### Short Form-Nepean Dyspepsia Quality of Life Index (SF-NDI)

SF-NDI total score and domain scores in the ITT population decreased (indicating an improvement) in both the tegaserod and placebo groups, from LSM scores of −39.9 and −36.4 (*P* = 0.102), respectively, at Day 50, to −39.0 and −37.8 (*P* = 0.537), respectively, at Day 71 (end of study; Table 4). There were no statistically significant differences in response between the two treatment groups.

**Table 4:** Effect of double-blind treatment on responses to the SF-NDI questionnaire (ITT population)

	Time point	Least squares mean PPI + tegaserod*	Least squares mean PPI + placebo*	Least squares mean treatment difference*	*P* value*	95% CI for treatment difference*
Total score	Day 50	−39.9	−36.4	−3.49	0.102	(−7.70, 0.71)
	Day 71 (EOS)	−39.0	−37.8	−1.21	0.537	(−5.13, 2.70)
Tension dimension score	Day 50	−7.5	−7.2	−0.34	0.489	(−1.33, 0.65)
	Day 71 (EOS)	−7.5	−7.3	−0.16	0.686	(−0.97, 0.64)
Interferences with daily activities	Day 50	−7.2	−6.6	−0.61	0.231	(−1.62, 0.40)
	Day 71 (EOS)	−7.1	−6.8	−0.27	0.538	(−1.13, 0.60)
Eating/drinking	Day 50	−9.1	−8.3	−0.81	0.066	(−1.67, 0.06)
	Day 71 (EOS)	−8.6	−8.5	−0.08	0.856	(−0.98, 0.81)
Knowledge/control	Day 50	−8.0	−7.3	−0.75	0.071	(−1.57, 0.07)
	Day 71 (EOS)	−7.9	−7.5	−0.45	0.322	(−1.36, 0.46)
Work/study	Day 50	−8.0	−7.1	−0.88	0.141	(−2.07, 0.30)
	Day 71 (EOS)	−7.8	−7.7	−0.11	0.824	(−1.13, 0.90)

* Adjusted mean treatment difference, *P* value and 95% CI are based on the least squares means in the PPI + tegaserod group minus the PPI + placebo group. Least squares means in either group were estimated by an analysis of covariance model that adjusted for center and baseline value.

EOS = end of study.

### Safety

Treatment with tegaserod during both the open-label, and double-blind phases was well tolerated, with few AEs and no serious AEs reported. The most common AE, diarrhea, was reported by 15 patients in the open-label phase (14.9%). In the double-blind phase, diarrhea was reported by three patients in the tegaserod group (8.1%) and none in the placebo group (Table 5).

**Table 5:** Summary of most commonly reported* AEs during open-label and double-blind phases (safety population†)

	Open-label phase	Double-blind phase	
Event	All patients (N = 101)	Tegaserod (N = 37)	Placebo (N = 33)
Any AE‡, n (%)	32 (31.7)	12 (32.4)	8 (24.2)
Diarrhea	15 (14.9)	3 (8.1)	0
Sinus congestion	1 (1.0)	2 (5.4)	1 (3.0)
Headache	4 (4.0)	2 (5.4)	0
Abdominal pain	2 (2.0)	1 (2.7)	1 (3.0)
Toothache	1 (1.0)	0	2 (6.1)
Constipation	2 (2.0)	0	1 (3.0)
Flatulence	2 (2.0)	1 (2.7)	0
Nausea	2 (2.0)	1 (2.7)	0
Migraine	2 (2.0)	0	0
Asthma	2 (2.0)	0	0
Hypertension	2 (2.0)	1 (2.7)	0

* Reported by ≥2 patients in any group.

† All patients who received at least one dose of study medication.

‡ Patients may have reported more than one AE.

Hypertension was the only cardiovascular-related AE reported in this study (n = 3), and was experienced by two patients in the open-label phase, and one patient in the double-blind phase (this patient had been randomized to tegaserod).

Although most AEs were “mild” or “moderate” in severity, six patients (5.9%) reported severe AEs during the open-label phase (three GI disorders, one migraine headache, one asthma and one arthralgia). During the double-blind phase, two patients in the tegaserod group (2.7%, bronchitis and GERD) and one patient in the placebo group (3.0%, abdominal pain) reported an AE of severe intensity.

AEs were generally considered by study investigators to be unrelated to study drug. Treatment-related AEs were reported for only three patients (8.1%) in the tegaserod group (abdominal pain, diarrhea, flatulence, nausea: one patient reported two AEs) and one patient (3.0%) in the placebo group (abdominal pain).

Nine patients discontinued from the open-label phase of the study due to AEs (the most frequent being diarrhea, n = 4, 4%). No patients discontinued treatment due to an AE during the double-blind phase. There were no clinically relevant changes in vital signs, blood chemistry, hematology, or ECG examinations and no patients died during the study.

## Discussion

In addition to their symptoms of dyspepsia (postprandial fullness, early satiety, and bloating) some functional dyspepsia patients also experience non-acid related heartburn (functional heartburn) [19]. The Rome Committee recommend that functional dyspepsia and functional heartburn are defined as distinct clinical entities; however, they also recognize that some overlap of symptoms exists [3,16]. While debate continues on the most appropriate definition to apply to functional disorders such as functional dyspepsia and functional heartburn, what is clear is that symptoms of both disorders overlap in some patients. Patients may also present with comorbidity with other GI disorders such as IBS and GERD. This observation is not surprising as recent studies have suggested that IBS and functional dyspepsia may share similar pathological mechanisms [20,21].

Treatments for patients with overlapping symptoms of functional dyspepsia and functional heartburn are currently suboptimal; while PPIs may be prescribed as first-line therapy in patients with symptoms of heartburn and dyspepsia, they have limited efficacy if patients’ symptoms are not acid-related [6]. Reasons for the lack of efficacy of PPIs in this patient group are unclear, but may be explained by the presence of dysmotility symptoms in patients with non-acid related symptoms or weakly acidic reflux that persists despite PPI therapy [22]. In addition, difficulties in designing clinical trials that account for the heterogeneity of functional dyspepsia and functional heartburn symptoms may have also hampered the development of effective treatments [23,24].

Tegaserod is a selective 5-HT_4_ agonist that has been shown to improve dysmotility symptoms in patients with IBS-C and chronic idiopathic constipation [7–11]. Additional studies have suggested it may also improve some dysmotility symptoms related to dyspepsia [15,25,26].

The women enrolled in this study were receiving PPI treatment for their symptoms of heartburn but without an adequate effect on their dyspeptic symptoms. A randomized withdrawal study design was selected in order to enrich the study population with patients who may respond to the active drug being tested, an approach that is considered to be particularly appropriate for drugs whose effect may be difficult to assess using other methodologies [27]. This approach was supported by the exploratory nature of the present study.

The results seen in this exploratory study, while not supportive, do not allow any definitive conclusions to be made on the role of tegaserod in women with overlapping functional dyspepsia and functional heartburn. One possible explanation could be that tegaserod, in line with the null hypothesis, may not be different to placebo in relieving the symptoms captured in this study, and in this patient population. Another explanation relates to a possible Type 2 error as the study (being exploratory) may not have had sufficient statistical power to demonstrate a benefit for tegaserod. The study design and sample size was based on practical reasons including the availability of resources and the limited timelines. Furthermore, specific limitations of a withdrawal study design may also have contributed to the negative outcome. In fact, it is conceivable that the combined effect of a relatively small sample size and a possible carry-over treatment effect from the open-label phase may mean that the study design was not sufficiently sensitive to detect differences between the two treatment groups. It is possible that extending the length of the double-blind portion of this study may have overcome any potential carry-over effect.

The patients’ quality of life scores (measured using the SF-NDI questionnaire) were consistent with the primary/secondary efficacy variables in that no treatment difference between tegaserod and placebo was observed, thus supporting the validity of the results. Treatment with tegaserod was well tolerated in this study, a finding that is consistent with previous studies in IBS-C, chronic idiopathic constipation and functional dyspepsia. Diarrhea was the most frequently reported AE, and as tegaserod is an agent with promotile activity in the GI tract, diarrhea is an expected treatment effect.

Since this study was completed, tegaserod was withdrawn from sale in the US due to a statistically significant imbalance in the incidence of cardiovascular ischemic events in patients taking tegaserod vs. placebo. Despite there being no access to tegaserod in normal clinical practice, we felt that publishing these data may benefit other researchers assessing the pharmacological activity of agents for treating this patient population.

In summary, while these results do not show that tegaserod relieved the symptoms of dyspepsia in these patients, no conclusive statement regarding treatment effect can be made from this pilot study. Further studies of functional dyspepsia patients with heartburn should employ a more rigorous and adequately powered parallel-group study design.
